# Physicochemical properties and toxic elements in bus stop dusts from Qingyang, NW China

**DOI:** 10.1038/s41598-018-30452-3

**Published:** 2018-08-22

**Authors:** Yongfu Wu, Xinwei Lu

**Affiliations:** 1 School of Geography and Tourism, Shaanxi Normal University, Xi’an, China; 2 College of Agriculture and Forestry Science and Technology, Longdong University, Qingyang, China

## Abstract

To appraise the content and pollution level of cadmium (Cd), arsenic (As), mercury (Hg), nickel (Ni) and lead (Pb) in bus stop dusts, representative samples (n = 53) were collected from the city of Qingyang in Gansu province, NW China. The Cd, As, Hg, Ni, and Pb contents and physicochemical properties (particle size, organic matter, pH and magnetic properties) of the bus stop dusts were investigated. Pollution levels were evaluated by the Nemero synthesis pollution index (NSPI) and geoaccumulation index (I_geo_). The results indicate that the magnetic susceptibilities of the bus stop dusts were higher than those in the local soils. Cd, As, Ni, and Pb contents ranged from 0.4 to 3.1, 7.1 to 16.3, 12.7 to 151.3, and 20.1 to 96.2 mg kg^−1^, with average values of 1.2, 10.1, 22.2, and 44.9 mg kg^−1^, while Hg content ranged from 4.5 to 1357.7 µg kg^−1^ with an average of 214.0 µg kg^−1^. The mean contents of Cd, As, Hg, Ni, and Pb were 12.0, 0.8, 10.0, 0.6, and 2.4 times the local soil background value, respectively. Cd, Hg and Pb in approximately 96%, 62% and 19% of the bus stop dusts were above the “moderately polluted” level in terms of I_geo_. As and Ni were defined as “practically unpolluted” in all of the bus stop dusts. The NSPI values of all of the bus stop dust samples exceeded 3, which reveals overall serious contamination of harmful elements.

## Introduction

The high contents of cadmium (Cd), arsenic (As), mercury (Hg), nickel (Ni) and lead (Pb) in bus stop dusts pose a potential threat to commuters and pedestrians. Cd, As, Hg, Ni and Pb persist in the environment, occurring in both inorganic and organic forms in nature, and are toxic to human health, flora and fauna^1–3^. Transportation, biomass burning, coal combustion in power plants, refining, waste incineration, and fossil fuel combustion are the main sources of these elements^4^. Fossil fuels (coal, fuel oil, etc.), which contain these toxic elements, were combusted in large quantities during the industrialization and urbanization of China and are still the main source of energy in the city of Qingyang in eastern Gansu province. The contents of Cd, As, Hg, Ni and Pb in the coal from North China are 0.1–3, 0.4–10, 0.2–0.5, 1–60, and 5–60 µg g^−1^, respectively^5^. The average contents of Cd, As, Hg, Ni and Pb in coal consumed in Gansu province are 0.1, 3.7, 0.2, 16.3, and 7.5 µg g^−1^, respectively^6^. Cd, As, Hg, Ni, Pb and other toxic elements contained in fossil fuels are discharged into the environment during combustion^7^. In 2012, the emissions of Cd, As, Hg, Ni and Pb from anthropogenic sources in China were approximately 527, 2529, 695, 3396, and 14398 t, respectively^6^. In 2013, the emissions of Cd, As, Hg, Ni and Pb from open municipal solid waste burning alone were estimated to be approximately 10, 132, 21, 44, and 97 in all of China and 0.2, 2.2, 0.4, 0.7, and 1.7 t in Gansu province, respectively^8^. Cd, As, Hg, Ni and Pb dispersed into the atmosphere and deposited as dusts in the water and soil through dry and wet deposition processes. The dust sources have an adverse effect on the atmosphere, water, soil, and human health.

The composition and quantities of toxic elements in street dusts are quality indicators of urban environments^9^. Street dusts can enter the human body by inhalation, ingestion and skin contact and may cause various health problems^10,11^. Numerous studies have been carried out on manganese, zinc, chrome and copper in dusts^12–17^. However, information on Cd, As, Hg, Ni and Pb in street dusts in moderate- or small-sized cities in Northwest China is very limited. Cd, As, Hg, Ni and Pb can affect the liver, skin, haematopoietic system, cardiovascular system, respiratory tract and gastrointestinal tract; represent a risk for bladder, lung, kidney, liver and skin cancer; and lead to black foot disease and Minamata disease^18–20^.

Since the development of modern cities, urban buses have become the main means of transportation for citizens due to their convenience and act as an “artery” for population mobility. However, research into the impact of pollution at bus stops on environmental and public health risks is rarely reported. The purpose of this study is to analyse the content of Cd, As, Hg, Ni and Pb in bus stop dusts from Qingyang and apply I_geo_ and NSPI to classify the contamination levels of Cd, As, Hg, Ni and Pb in different bus stop dusts. These results could be used to establish layouts and quantities of bus stops and formulate appropriate environmental management and planning policies according to bus stop pollution, population and traffic volume, production and living conditions by the municipal department. Our government and authorities could reasonably plan locations and quantities of bus stops, bus routes, etc.; adopt mitigation policies; and control measures to better maintain the “blue sky plan” in the Qingyang region.

## Results and Discussion

### Physicochemical properties of bus stop dusts

Table 1 lists the physicochemical properties of bus stop dusts from Qingyang, NW China. The pH values ranged from 6.8 to 12.0 (with an average of 9.0), which were higher than the pH of the local soil (8.3)^21^. The low-frequency magnetic susceptibility (χ_LF_) indicates the relative content of magnetic minerals^22^ and varied from 52 × 10^−8^ to 792 × 10^−8^ m^3^ kg^−1^, with an average of 213 × 10^−8^ m^3^ kg^−1^. These values are lower than the magnetic susceptibility of street dusts in Lanzhou (450 × 10^−8^ m^3^ kg^−1^)^23^, Xi’an (487 × 10^−8^ m^3^ kg^−1^)^24^, Beijing Olympic Park (243 × 10^−8^ m^3^ kg^−1^)^25^, Ezhou (4150 × 10^−8^ m^3^ kg^−1^), Zhuzhou (663 × 10^−8^ m^3^ kg^−1^), Hezhang (527 × 10^−8^ m^3^ kg^−1^)^26^, and Loudi (880 × 10^−8^ m^3^ kg^−1^)^27^ and higher than the magnetic susceptibility of street dusts in Wuhan (132 × 10^−8^ m^3^ kg^−1^)^28^, Seoul (62 × 10^−8^ m^3^ kg^−1^)^29^, and Plovdiv (193 × 10^−8^ m^3^ kg^−1^)^30^. The frequency-dependent magnetic susceptibility (χ_FD_) indicates the relative content of superparamagnetic minerals^23^ and varied from 0.1% to 13.5%, with an average of 3.0%. These values are lower than the χ_FD_ of street dusts in Hezhang (4.4%) and Zhuzhou (3.3%)^26^ and higher than the χ_FD_ of street dusts in Wuhan (1.0%)^28^, Beijing Olympic Park (1.6%)^25^, Lanzhou (2.2%)^23^, and Ezhou (2.9%)^26^.

**Table 1:** Cd, As, Hg, Ni and Pb contents and the physical and chemical properties of bus stop dusts from Qingyang.

SN	1	2	3	4	5	6	7	8	9	10	11	12	13	14	15	16	17	18	19	20	21	22	23	24	25	26	27
pH	8.1	8.3	8.0	8.6	8.0	12.0	11.1	10.8	10.4	10.1	9.9	9.8	9.8	9.4	6.8	7.5	8.2	8.5	8.4	7.4	9.0	8.0	8.5	8.8	9.0	9.1	9.1
OM (%)	18.7	6.9	9.9	10.3	15.9	4.8	12.0	12.3	15.8	10.0	2.8	6.9	11.8	8.0	8.5	10.5	7.3	11.5	12.9	9.9	9.9	17.4	15.0	13.7	7.0	18.8	8.7
PM < 2.5 (%)	9.8	6.3	6.7	11.2	9.7	4.1	3.9	3.4	10.3	4.1	2.1	2.1	2.9	9.5	5.8	6.1	6.7	7.0	2.7	3.5	4.5	5.5	4.0	2.9	8.7	6.5	7.5
2.5 < PM < 10 (%)	26.3	23.1	20.3	35.8	14.0	7.7	13.1	12.1	23.2	10.5	6.9	5.0	8.9	31.0	20.6	17.2	14.0	18.0	18.0	12.0	18.7	11.0	19.1	22.3	24.0	25.0	22.0
10 < PM < 50 (%)	56.4	52.3	58.4	46.5	39.5	28.4	42.7	36.2	53.7	32.0	28.6	34.6	38.3	47.6	58.7	43.6	43.0	42.2	34.0	35.0	37.0	38.0	42.0	41.0	40.0	42.5	41.5
PM > 50 (%)	7.6	18.3	14.6	6.5	36.9	59.8	40.4	48.2	12.8	53.4	62.4	58.3	49.9	11.9	14.9	33.1	36.3	32.9	45.4	49.6	39.8	45.5	34.9	33.8	27.4	26.0	29.0
χ_LF_ (10^−8^ m^3^ kg^−1^)	201.9	197.0	116.7	521.7	198.9	347.2	269.7	180.1	279.4	51.9	138.1	191.4	222.4	141.2	208.7	150.6	236.0	145.3	122.9	234.4	162.4	201.8	273.0	131.1	203.1	173.8	174.3
χ_HF_ (10^−8^ m^3^ kg^−1^)	199.1	183.8	107.7	516.3	192.7	340.0	266.1	172.1	276.9	48.9	135.9	186.7	215.7	138.6	180.5	139.0	234.4	142.1	117.4	233.3	160.0	186.4	272.3	128.6	191.1	170.7	174.2
χ_FD_ (%)	1.4	6.7	7.7	1.0	3.1	2.1	1.3	4.4	0.9	5.8	1.6	2.5	3.0	1.8	13.5	7.7	0.7	2.2	4.5	0.5	1.5	7.6	0.3	1.9	5.9	1.8	0.1
Hg (µg kg^−1^)	116.4	243.4	194.6	35.8	71.3	217.0	33.3	466.5	326.6	139.3	116.1	240.4	361.7	108.2	514.6	1357.7	218.0	75.0	149.5	164.3	189.1	223.5	134.0	90.0	906.0	235.4	88.3
As (mg kg^−1^)	11.8	11.9	12.5	9.2	11.1	11.0	8.8	9.8	10.4	9.6	8.7	9.1	10.5	11.1	8.8	10.3	16.3	8.5	13.7	11.1	9.1	9.4	9.8	9.1	8.7	9.5	8.9
Cd (mg kg^−1^)	1.8	3.1	1.5	1.0	1.1	1.0	1.0	0.9	1.8	1.2	0.7	0.9	1.0	1.7	1.4	2.3	1.6	0.7	1.5	1.3	1.9	1.7	0.8	0.8	0.9	0.8	1.2
Ni (mg kg^−1^)	20.9	23.3	22.8	18.9	20.4	18.6	15.6	25.2	31.0	17.4	16.5	19.9	21.4	28.2	151.3	24.2	17.0	15.3	21.8	20.5	21.4	17.7	16.4	18.2	15.1	16.2	17.1
Pb (mg kg^−1^)	73.8	62.3	46.4	65.3	44.1	32.6	31.3	33.5	34.2	40.9	38.3	34.2	39.7	47.5	40.8	40.5	49.1	36.1	53.0	60.2	52.7	37.8	32.5	36.1	32.3	56.5	36.0
Hg I_geo_	2.3	3.3	3.0	0.6	1.6	3.2	0.5	4.3	3.8	2.5	2.3	3.3	3.9	2.2	4.4	5.8	3.2	1.6	2.6	2.8	3.0	3.2	2.5	1.9	5.2	3.3	1.9
Hg PI	5.8	12.2	9.7	1.8	3.6	10.9	1.7	23.3	16.3	7.0	5.8	12.0	18.1	5.4	25.7	67.9	10.9	3.7	7.5	8.2	9.5	11.2	6.7	4.5	45.3	11.8	4.4
As I_geo_	−0.6	−0.6	−0.5	−1.0	−0.7	−0.7	−1.1	−0.9	−0.8	−0.9	−1.1	−1.0	−0.8	−0.7	−1.1	−0.8	−0.2	−1.1	−0.4	−0.7	−1.0	−1.0	−0.9	−1.0	−1.1	−0.9	−1.0
As PI	1.0	1.0	1.0	0.8	0.9	0.9	0.7	0.8	0.9	0.8	0.7	0.7	0.9	0.9	0.7	0.8	1.3	0.7	1.1	0.9	0.7	0.8	0.8	0.7	0.7	0.8	0.7
Cd I_geo_	3.4	4.2	3.2	2.5	2.8	2.5	2.6	2.4	3.4	2.8	2.1	2.4	2.6	3.3	3.1	3.7	3.2	2.1	3.1	2.9	3.5	3.4	2.2	2.2	2.4	2.3	2.8
Cd PI	31.3	38.1	28.8	22.8	25.2	22.9	23.2	22.2	31.3	25.5	18.9	21.8	23.2	30.4	27.8	34.0	29.2	19.5	28.4	26.8	31.7	30.7	20.1	20.4	21.4	21.2	25.7
Ni I_geo_	−1.3	−1.2	−1.2	−1.5	−1.4	−1.5	−1.8	−1.1	−0.8	−1.6	−1.7	−1.4	−1.3	−0.9	1.5	−1.1	−1.6	−1.8	−1.3	−1.4	−1.3	−1.6	−1.7	−1.5	−1.8	−1.7	−1.6
Ni PI	0.6	0.7	0.6	0.5	0.6	0.5	0.4	0.7	0.9	0.5	0.5	0.6	0.6	0.8	4.3	0.7	0.5	0.4	0.6	0.6	0.6	0.5	0.5	0.5	0.4	0.5	0.5
Pb I_geo_	1.4	1.1	0.7	1.2	0.6	0.2	0.2	0.2	0.3	0.5	0.4	0.3	0.5	0.8	0.5	0.5	0.8	0.4	0.9	1.1	0.9	0.4	0.2	0.4	0.2	1.0	0.4
Pb PI	3.9	3.3	2.5	3.5	2.3	1.7	1.7	1.8	1.8	2.2	2.0	1.8	2.1	2.5	2.2	2.2	2.6	1.9	2.8	3.2	2.8	2.0	1.7	1.9	1.7	3.0	1.9
**SN**	**28**	**29**	**30**	**31**	**32**	**33**	**34**	**35**	**36**	**37**	**38**	**39**	**40**	**41**	**42**	**43**	**44**	**45**	**46**	**47**	**48**	**49**	**50**	**51**	**52**	**53**	**GS** ^43^
pH	9.2	8.9	8.8	9.1	8.4	8.6	9.3	9.1	8.9	9.6	9.4	9.5	8.7	8.8	8.5	8.7	9.7	8.6	8.6	9.4	8.8	9.1	8.8	8.7	9.6	8.8	8.5
OM (%)	16.3	5.0	8.4	9.5	7.5	9.9	15.3	8.7	6.8	16.5	17.7	7.8	17.8	11.9	12.9	7.4	15.5	8.9	17.5	9.4	11.5	16.8	12.7	16.3	5.8	17.5	2.7
PM < 2.5 (%)	7.0	7.6	5.5	10.6	9.7	6.2	3.5	8.4	6.7	5.5	7.4	5.5	8.7	6.3	4.4	5.8	4.6	5.3	11.3	7.0	7.3	5.4	3.3	2.8	3.5	4.9	#
2.5 < PM < 10 (%)	18.0	17.0	16.0	15.0	14.0	15.0	16.0	11.0	14.0	17.0	18.0	9.0	13.0	14.0	12.0	15.0	29.0	15.0	27.0	11.0	25.0	24.0	33.0	28.0	32.0	17.0	#
10 < PM < 50 (%)	42.2	35.5	36.9	38.5	39.5	41.3	42.6	45.6	43.0	31.9	36.8	43.9	40.7	35.1	52.0	54.0	50.0	35.0	34.0	25.0	24.0	28.0	29.0	8.0	17.0	18.0	#
PM > 50 (%)	32.9	39.9	41.6	35.9	36.9	37.4	37.9	35.0	36.3	45.6	37.8	41.7	37.6	44.6	31.7	25.3	16.4	44.7	27.7	57.1	43.8	42.6	34.7	61.2	47.5	60.1	#
χ_LF_ (10^−8^ m^3^ kg^−1^)	108.7	209.1	207.4	159.6	135.6	138.4	196.1	252.7	392.8	144.9	186.9	165.7	156.6	178.8	792.4	286.0	284.3	344.3	230.1	164.8	274.5	195.9	179.5	220.1	199.3	170.7	#
χ_HF_ (10^−8^ m^3^ kg^−1^)	105.7	190.6	205.2	156.8	133.0	136.9	192.2	247.4	368.9	136.6	178.4	162.2	151.5	178.2	770.9	284.7	282.0	340.6	229.0	157.7	269.4	187.7	176.3	201.0	198.0	167.8	#
χ_FD_ (%)	2.8	8.8	1.1	1.8	1.9	1.1	2.0	2.1	6.1	5.7	4.5	2.1	3.3	0.3	2.7	0.5	0.8	1.1	0.5	4.3	1.9	4.2	1.8	8.7	0.7	1.7	#
Hg (µg kg^−1^)	31.6	57.2	445.3	138.9	894.2	85.2	176.8	70.1	87.3	175.5	66.0	47.9	99.7	28.5	675.9	110.5	5.1	443.5	8.0	19.3	233.7	4.5	117.1	42.2	203.0	36.3	20.0
As (mg kg^−1^)	10.6	11.3	9.4	11.9	10.3	9.0	8.2	10	12.3	11.9	9.7	8.3	7.1	9.6	13.2	9.1	10.2	10	8.2	9.9	10.1	9.6	8.7	8.6	9.4	9.5	12.6
Cd (mg kg^−1^)	0.9	0.9	1.0	1.0	0.8	0.8	1.3	0.4	1.3	1.4	0.9	1.0	1.2	1.0	2.2	1.3	1.0	2.0	0.7	1.1	1.5	1.3	1.9	0.9	1.5	0.6	0.1
Ni (mg kg^−1^)	18.0	19.8	15.5	18.6	16.5	20.9	14.8	18.9	32.2	28.8	15.5	17.4	13.5	19.0	19.5	17.1	17.9	51.3	12.7	19.1	17.9	16.9	15.3	14.6	19.4	12.7	35.2
Pb (mg kg^−1^)	33.7	31.5	38.4	39.7	32.8	43.9	43.6	30.8	95.4	96.2	34.8	35.3	39.9	39.8	76.3	71.9	34.4	62.6	34.4	43.8	45.3	49.3	37.9	33.8	46.7	20.1	18.8
Hg I_geo_	0.4	1.3	4.2	2.5	5.2	1.8	2.9	1.5	1.9	2.9	1.5	1.0	2.1	0.3	4.8	2.2	−2.2	4.2	−1.6	−0.3	3.3	−2.4	2.3	0.8	3.1	0.6	−0.3
Hg PI	1.6	2.9	22.3	6.9	44.7	4.3	8.8	3.5	4.4	8.8	3.3	2.4	5.0	1.4	33.8	5.5	0.3	22.2	0.4	1.0	11.7	0.2	5.9	2.1	10.2	1.8	1
As I_geo_	−0.8	−0.7	−1.0	−0.6	−0.8	−1.0	−1.2	−0.9	−0.6	−0.6	−0.9	−1.1	−1.4	−0.9	−0.5	−1.0	−0.8	−0.9	−1.2	−0.9	−0.9	−0.9	−1.1	−1.1	−1.0	−0.9	−0.6
As PI	0.9	0.9	0.8	1.0	0.8	0.7	0.7	0.8	1.0	1.0	0.8	0.7	0.6	0.8	1.1	0.7	0.8	0.8	0.7	0.8	0.8	0.8	0.7	0.7	0.8	0.8	1.0
Cd I_geo_	2.4	2.4	2.5	2.5	2.2	2.2	2.9	1.3	3.0	3.0	2.5	2.5	2.8	2.5	3.7	2.9	2.6	3.5	2.1	2.7	3.2	3.0	3.5	2.5	3.2	1.8	−0.6
Cd PI	22.1	21.7	23.1	23.1	19.6	19.8	26.3	11.5	27.0	27.7	22.6	22.9	25.4	23.0	33.9	26.7	23.2	32.2	18.8	24.4	28.8	26.9	31.9	22.4	28.9	16.1	−5.6
Ni I_geo_	−1.6	−1.4	−1.8	−1.5	−1.7	−1.3	−1.8	−1.5	−0.7	−0.9	−1.8	−1.6	−2.0	−1.5	−1.4	−1.6	−1.6	0.0	−2.1	−1.5	−1.6	−1.6	−1.8	−1.9	−1.4	−2.1	−2.1
Ni PI	0.5	0.6	0.4	0.5	0.5	0.6	0.4	0.5	0.9	0.8	0.4	0.5	0.4	0.5	0.6	0.5	0.5	1.5	0.4	0.5	0.5	0.5	0.4	0.4	0.6	0.4	0.4
Pb I_geo_	0.3	0.2	0.4	0.5	0.2	0.6	0.6	0.1	1.8	1.8	0.3	0.3	0.5	0.5	1.4	1.4	0.3	1.2	0.3	0.6	0.7	0.8	0.4	0.3	0.7	−0.5	−0.5
Pb PI	1.8	1.7	2.0	2.1	1.7	2.3	2.3	1.6	5.1	5.1	1.9	1.9	2.1	2.1	4.1	3.8	1.8	3.3	1.8	2.3	2.4	2.6	2.0	1.8	2.5	1.1	1.1

SN: Sample number; GS: Background value of Gansu soil; OM: Organic matter; PM: Particulate matter.

SN: Sample number; GS: Background value of Gansu soil; OM: Organic matter; PM: Particulate matter; ^#^Not available.

The values of χ_FD_ and χ_LF_ of road dusts can reflect the effect of anthropogenic activities^23^. Table 1 shows that bus stop dusts from Qingyang had the characteristics of higher χ_LF_ and lower χ_FD_. The dust samples collected from bus stops in areas with a high volume of vehicles and dense population, such as bus stations, motor vehicle maintenance service areas, schools, and parks, had larger χ_LF_ values, likely due to the combined result of a high density of vehicles, oil varnish, lubricating oil, and other production and operating activities near these bus stops. The dust samples collected from bus stops in areas with a low density of vehicles, such as residential areas and hotels, had small χ_LF_ values, likely due to the low volume of vehicles and few other sources and operating activities near these bus stops.

The organic matter (OM) content in the bus stop dust samples ranged from 2.8% to 18.8%, with a mean value of 11.5%. The dust samples collected from bus stops near schools, parks, hotels and residential areas had a higher OM content.

The particle size analysis results indicate that the proportion of PM >50, PM 10–50, PM 2.5–10 and PM <2.5 in the bus stop dusts from Qingyang ranged from 6.5% to 62.4%, 8.0% to 58.7%, 5.0% to 35.8% and 2.1% to 11.3%, with averages of 37.0%, 38.9%, 18.0% and 6.1%, respectively. Particulate matter with diameters below 100 µm (PM 100) can be transported by suspension, while particulate matter with diameters below 10 µm (PM 10) can remain airborne over a long distance and for a long time^31^. Airborne PM 2.5 and PM 10 can seriously affect human health, as they can directly enter the respiratory system through inhalation. In addition, the sampling method used can result in a loss of particles, thus altering the original particle size fractionation. Therefore, the mean values indicate that approximately one-fourth of bus stop dusts could potentially enter the respiratory system (PM ≤ 10 comprises 24.1%), and resuspended PM (PM ≤ 50) in the atmosphere may comprise more than 63.0% of all bus stop dusts. Therefore, the Qingyang bus stop dusts have a large potential to degrade atmospheric quality and cause harm to human health.

### Contents of Cd, As, Hg, Ni and Pb in bus stop dusts

**Figure 1:**
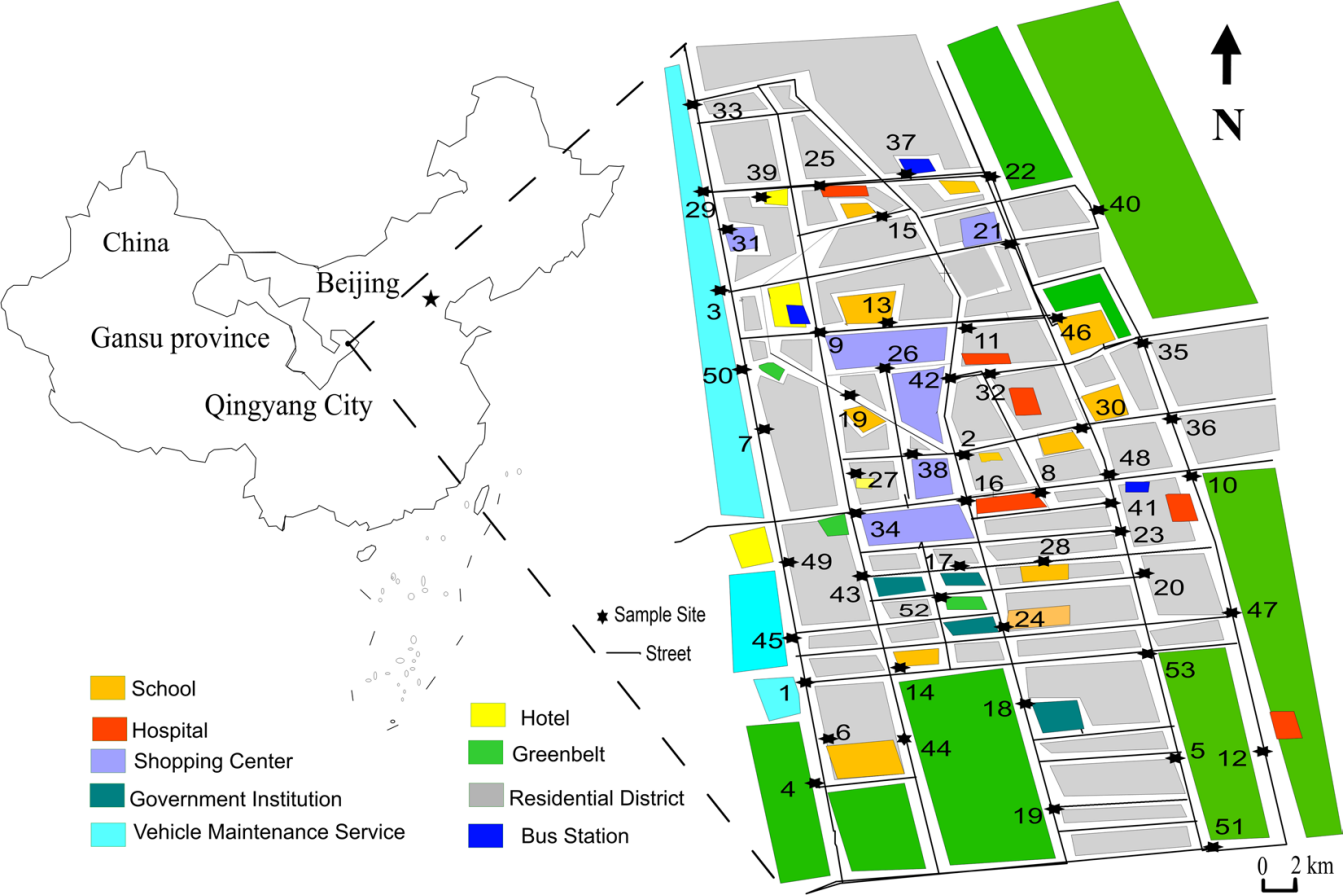
Sampling sites and study area in Qingyang, China (the background map was made using surfer 11.0).

The mean content of Cd in the bus stop dusts is 12.0 times the background Cd content in Gansu soil (GS). The Cd contents in all of the bus stop dust samples are 4.0 to 31.0 times the Cd content in GS (0.1 mg kg^−1^) (Table 2). The dust samples from bus stops near shopping centres (samples 21 and 42), schools (samples 2, 14, and 17), hospitals (sample 16), vehicle maintenance services (samples 1 and 45), bus stations (sample 9), government institutions (sample 22) and so on (Fig. 1) had higher Cd contents (above average content), likely as a result of the large population and vehicle flow, lubricating oil, other production and operating activities^3,4,6^ near these bus stops. Lower Cd contents were obtained in dust samples collected at bus stops near residential districts (samples 35 and 53) and the greenbelt (sample 18) (Fig. 1), possibly as a result of there being few other production and operating activities near these bus stops.

**Table 2:** Cd, As, Hg, Ni and Pb contents in bus stop dusts from Qingyang and other cities reported in the literature.

Heavy metal	Location/Ref.	Mean	Max	Min
Cd (mg kg^−1^)	Guiyang, China/^44^	0.58	1.17	0.04
	Chongqing, China/^45^	5.0	19.2	0.9
	Avilés, Spain/^46^	45.1	139.0	11.0
	Luanda, Angola/^47^	1.1	4.0	0.7
	Qingyang, China/this study	1.2	3.1	0.4
As (mg kg^−1^)	Guiyang, China/^44^	11.3	19.2	7.2
	Chongqing, China/^45^	6.8	15.5	2.3
	Avilés, Spain/^46^	37.6	110.1	16.4
	Luanda, Angola/^47^	5.0	7.8	3.5
	Qingyang, China/this study	10.1	16.3	7.1
Hg (µg kg^−1^)	Guiyang, China/^44^	330	1120	80
	Chongqing, China/^45^	110	330	30
	Avilés, Spain/^46^	2930	10300	900
	Luanda, Angola/^47^	130	570.0	30
	Qingyang, China/this study	214.0	1357.7	4.5
Ni (mg kg^−1^)	Guiyang, China/^44^	61.1	146.0	32.1
	Chongqing, China/^45^	22.2	33.8	10.5
	Avilés, Spain/^46^	43.3	69.3	22.4
	Luanda, Angola/^47^	10.0	32	6.2
	Qingyang, China/this study	22.2	151.3	12.7
Pb (mg kg^−1^)	Guiyang, China/^44^	67.8	356.0	24.6
	Chongqing, China/^45^	75.6	148.8	37.3
	Avilés, Spain/^46^	496.0	1482.0	208
	Luanda, Angola/^47^	351.0	1856.0	74.0
	Qingyang, China/this study	44.9	96.2	20.1

The content of Hg in bus stop dusts from Qingyang ranged from 4.5 to 1357.7 µg kg^−1^, with a mean of 214.0 µg kg^−1^ (Table 2). The mean content of Hg in the bus stop dusts is over 10.0 times higher than the Hg content of GS (20.0 µg kg^−1^). Most of the dust samples from bus stop near hospitals (samples 8, 16, 25, and 32) and shopping centres (samples 9, 16, and 42) (Fig. 1) had higher Hg contents (above average content). These bus stops are likely to be locations with high vehicle density, decoration, mercury thermometers and sphygmomanometers^4,6,7^. Lower Hg contents were obtained in dust samples from bus stops in areas with low traffic volume, such as residential communities (samples 41, 47, 51, and 53), schools (samples 4, 28, 44, and 46), and hotels (samples 39 and 49) (Fig. 1). These bus stops have a low density of vehicles but a large amount of coal burning, building and infrastructure construction development.

The content of Pb in bus stop dusts from Qingyang ranged from 20.1 to 96.2 mg kg^−1^, with a mean of 44.9 mg kg^−1^ (Table 2). The mean content of Pb in the bus stop dusts is 2.4 times larger than the Pb content in GS (18.8 mg kg^−1^). Most of the dust samples from bus stops near bus stations (sample 37), residential areas (sample 36), vehicle maintenance service sites (sample 1) and shopping centres (sample 42) had higher Pb contents (Fig. 1). These bus stops are likely to be locations of high vehicle density along with a large amount of coal burning, building and infrastructure construction development, decoration and so on. Lower Pb contents were found in dust samples from bus stops near city and countryside intersections and city margins, such as samples 7, 29, 35 and 53 (Fig. 1). These bus stops are located in areas with a lower population, reduced traffic flow and reduced human activity.

Table 2 shows that the As and Ni contents in the bus stop dusts ranged from 7.1 to 16.3 and 12.7 to 151.3 mg kg^−1^, with mean values of 10.1 and 22.2 mg kg^−1^, respectively. The mean contents of As and Ni in most bus stop dusts were less than the contents of As (12.6 mg kg^−1^) and Ni (35.2 mg kg^−1^) in GS. These results show that most bus stop dust samples contain small amounts of As and Ni.

Table 2 shows the contents of Cd, As, Hg, Ni and Pb in Qingyang bus stop dusts compared with those from other cities. The mean content of Cd in bus stop dusts from Qingyang (NW China) are lower than those in road dusts from Chongqing (SW China) and Avilés (Spain) and higher than those in road dusts from Guiyang (SW China) and Luanda (Angola). The results show that Cd pollution in the streets of Qingyang may be lower than that in Chongqing and Avilés and higher than that in Guiyang and Luanda. The mean contents of As, Ni and Hg in bus stop dusts from Qingyang are lower than those in road dusts from Guiyang and Avilés. Thus, As, Ni and Hg pollution in the streets of Qingyang may be lower than that in Chongqing and Avilés and higher than that in Guiyang and Luanda. The mean contents of As and Hg in bus stop dusts from Qingyang are higher than those in road dusts from Chongqing and Luanda. Thus, As and Hg pollution in the streets of Qingyang may be higher than that in Chongqing and Luanda. The mean content of Ni in bus stop dusts from Qingyang is equal to that in road dusts from Chongqing. Thus, Ni pollution in the streets of Qingyang may be equal to that in Chongqing. The mean Pb content in bus stop dusts from Qingyang is lower than those found in the four cities mentioned above. Thus, Pb pollution in the streets of Qingyang may be lower than that in the four cities mentioned above.

### Cd, As, Hg, Ni and Pb pollution assessment

**Figure 2:**
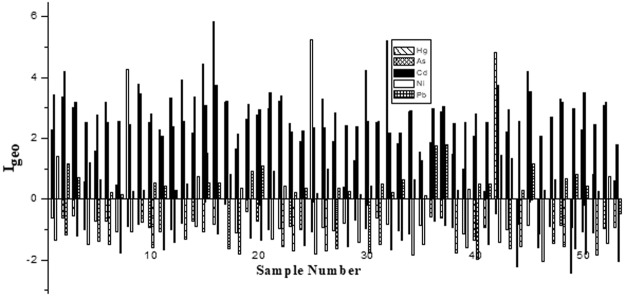
I_geo_ of Cd, As, Hg, Ni and Pb in bus stop dusts from Qingyang.

Table 1 presents the I_geo_ results for Cd, As, Hg, Ni and Pb in the Qingyang bus stop dusts. The values of I_geo_ for Cd, As, Hg, Ni and Pb ranged from 1.3 to 4.2, −1.4 to −0.2, −2.4 to 5.8, −2.1 to 1.5 and −0.5 to 1.8, with averages of 2.8, −0.9, 2.3, −1.4 and 0.6, respectively. Figure 2 shows that most of the bus stop dust samples could not be classified as polluted by As and Ni. The average I_geo_ and approximately 64% of the I_geo_ values for Cd were between 2 and 3, indicating that the bus stop dusts were “moderately to strongly” polluted with Cd, while approximately 32% of the I_geo_ values for Cd were over 3, revealing that the bus stop dusts were “strongly” polluted. The average I_geo_ and approximately 26% of the I_geo_ values for Hg were between 2 and 3, indicating that the bus stop dusts were “moderately to strongly” polluted with Hg, while approximately 36% of the I_geo_ values for Hg were over 3, revealing that the bus stop dusts were “strongly” polluted. The average I_geo_ and approximately 77% of the I_geo_ values for Pb were between 0 and 1, indicating that the bus stop dusts were “slightly to moderately” polluted with Pb, while approximately 19% of the I_geo_ values for Pb were between 1 and 2, revealing that the bus stop dusts were “moderately” polluted.

**Figure 3:**
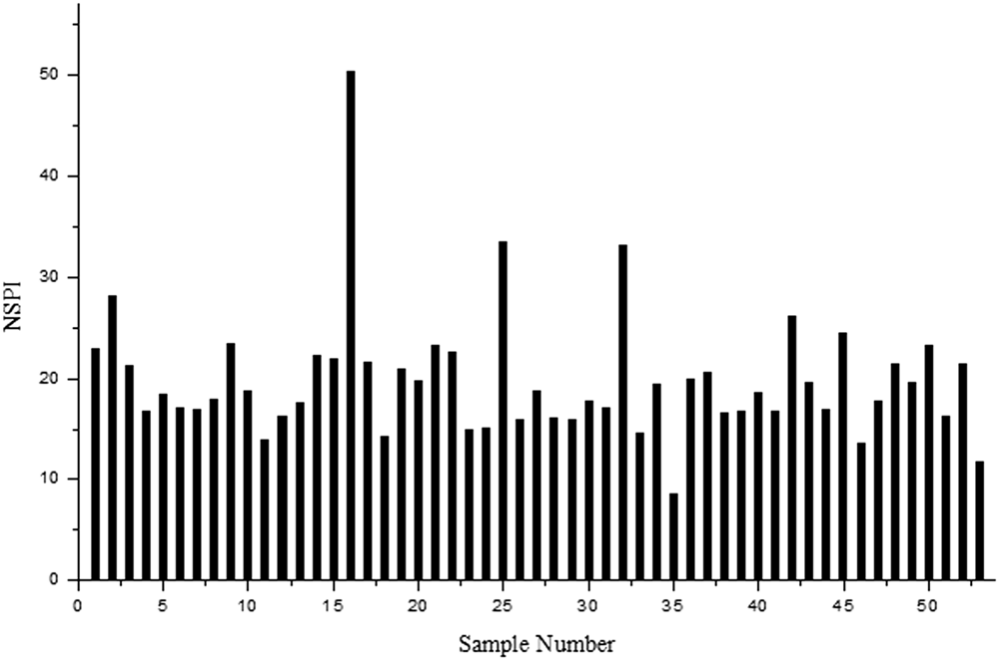
NSPI of bus stop dusts from Qingyang.

The results of the Nemero synthesis pollution index (NSPI) analysis of Cd, As, Hg, Ni and Pb in the bus stop dust samples are shown in Fig. 3. The NSPI values for all bus stop dust samples varied from 9.0 to 50.3, with a mean of 19.8. The NSPI values for all bus stop dust samples exceeded 3, which reveals serious contamination by harmful toxic elements overall.

## Conclusions

Cd, As, Hg, Ni and Pb pollution and the physicochemical properties of bus stop dusts from Qingyang were studied. The pH of the bus stop dusts is slightly higher than the background value of Gansu soil. Most bus stop dusts have the characteristics of lower χ_FD_ and higher χ_LF_, which reflect the frequency of anthropogenic activities. Resuspended particulate matter comprises over 63% of the bus stop dusts, and about one-fourth of the bus stop dusts have the potential to directly enter the respiratory system. The geoaccumulation index indicates that approximately 96%, 93% and 19% of the bus stop dusts are more than “moderately polluted” by Cd, Hg and Pb, respectively, while most of the bus stop dusts could not be classified as polluted by As and Ni. The prime anthropogenic sources of Cd, Hg and Pb in bus stop dusts from Qingyang are likely to be the high vehicle density, lubricating oil, welding activities, mercury thermometers and sphygmomanometers, large population and vehicle flow, building and infrastructure construction development, decoration, and so on. The NSPI values reveal overall serious contamination by toxic elements. Therefore, our government and agencies could ameliorate pollution at bus stops in Qingyang by considering vehicle and population flow, production and living conditions, and authorities should plan reasonable locations and quantities of bus stops, bus routes, etc. and adopt mitigation policies and control measures to better maintain the “blue sky plan” in the Qingyang region.

## Materials and Methods

### Survey region

The city of Qingyang (35°15′–37°10′N and 106°20′–108′45′E) is situated in the Loess Plateau region in eastern Gansu province (Fig. 1). The city spans over 996.4 km^2^, with a total population of approximately 325.2 thousand in 2015. The city has a characteristic semi-arid temperate continental monsoon climate with the following annual averages: evaporation capacity of 1613.1 mm, precipitation capacity of 465.7 mm, temperature of 9.6 °C, wind velocity of 2.7 m s^−1^, and sunlight of 2490.4 h. The moisture content in the local soil is 11.7%, and the electrical conductivity is 92.4 s cm^−1^. The texture analysis indicates that the soil is composed of 8.1% clayey soil, 39.5% silty sand and 52.4% fine sand^21^.

Approximately 100 thousand motor vehicles were driven in Qingyang in 2015. Qingyang has abundant oil, gas, and coal resources and was established as one of the important industrial bases of China by the state council in 2010. Oil exploitation, refining and transport are the major industries in Qingyang. Historically, Qingyang was an important agriculture city in NW China. At present, it is experiencing rapid urbanization and industrialization, making it a typical representative of the rapidly developing small- and medium-sized cities in NW China.

### Samples and analytical procedures

Fifty-three samples were taken in Qingyang (Fig. 1). The samples came from various types of bus stops, including those near schools, areas with heavy and low traffic density, residential districts, and commercial areas. Bus stop dusts were collected as composite samples of approximately 500 g at every sampling site in April, 2016. The samples were swept up by a plastic brush and tray from five to seven points at each platform and the surrounding area of the bus stop. The sample areas for each bus stop were approximately 5 m wide ×10 m long. All 53 composite dust samples were collected in the same manner. The dust samples were then packed in sealed plastic bags, labelled and transported to the lab. All the samples were sieved through a 20-mesh nylon sieve to remove large stones and plant debris and then air-dried in the lab for 14 days. To prevent contamination, all processing procedures were carried out without contact with any ironware, and the sieve was wiped by water-free ethanol-soaked absorbent cotton between samples.

High-frequency (χ_HF_, 4.7 kHz) and low-frequency signals (χ_LF_, 0.47 kHz) were measured by a Bartington magnetic susceptibility meter (MS2, Bartington Instruments Ltd., Oxford, UK). The frequency-dependent susceptibility (χ_FD_) was calculated as χ_FD_ = (χ_LF_ − χ_HF_)/χ_LF_ × 100^32,33,48^. The pH was measured in a 2:1 mixture of ultrapure water and sample^34^. In addition, the particulate matter (PM) was measured by a laser analyser (Microtrac X100 & S3500, America). The organic matter (OM) was measured by the loss-on-ignition method of Xie^33^. Samples were oven-dried at 105 °C, weighed and then ignited in a muffle furnace at 400 °C for 4 h before being re-weighed to determine the OM loss.

Each sample (0.5 g) was digested in a mixture of HNO_3_-HCl-HF (5 ml HNO_3_, 2 ml HCl and 1 ml HF) in a polytetrafluoroethylene (PTFE) pipe. The PTFE pipe was covered and placed in a microwave digestion system at 180 °C for 1 h. The contents of As and Hg in the bus stop dust samples were determined by double channel atomic fluorescence spectrometry^35^. The content of Cd in the bus stop dust samples was determined by graphite furnace atomic absorption spectroscopy^36^. The total concentrations of Pb and Ni were determined by flame atomic absorption spectroscopy (FAAS, Analytik Jena AG, ZEEnit 700P)^35^.

Analytical-grade reagents and chemicals, purchased from Tianjin Kemiou Chemical Reagent Co., Ltd. (China), were used for sample analysis. All solutions were prepared using ultrapure water from a Milli-Q System (Pall Company, UK). All glassware and plastics were soaked in HNO_3_ (8%) for at least 20 h and rinsed three times with tap water, secondary deionized water and ultrapure water, respectively. Quality control (QC) samples were a standard soil substance (GBW07406-GSS-6) and standard reagents (i.e., 0.5 and 5 μg ml^−1^) and were injected after every 18 samples to assess the stability of the instrument. The variations in the metal concentrations of the QCs were <13%. Reagent blanks were also included in each batch of analyses to check for any cross contamination of the different digested samples. The precision of the analytical procedures was expressed as the relative standard deviation (RSD), which ranged from 3% to 8% and was calculated from the standard deviation divided by the average value. Calibration curves were prepared separately for each element using different concentrations (i.e., 0.1, 0.25, 0.5, 1, 2.5, 5 and 10 μg ml^−1^) of the standard solutions. The instrument was set to zero concentration for all samples using a reagent blank. Each determination was based on the mean value of three replicate measurements, the detection limits of Cd, As, Hg, Ni and Pb are 0.1 µg kg^−1^, 13 µg kg^−1^, 3 µg kg^−1^, 0.3 µg kg^−1^, 0.06 mg kg^−1^, respectively. The statistics were computed using Origin 8.0 software and Microsoft Office Excel 2016.

### Pollution assessment methods

There are numerous calculation methods for quantifying the degree of harmful element enrichment or contamination in dusts and soils^37–40^. The geoaccumulation index (I_geo_) and Nemero synthesis pollution index (NSPI) are used frequently as environment evaluation methods. Therefore, assessment of the element pollution levels in the bus stop dusts used both I_geo_ and NSPI.

The following equation was used to calculate the results:

1 $${{\rm{I}}}_{{\rm{geo}}}={\mathrm{log}}_{2}\frac{{{C}}_{{\rm{n}}}}{1.5{B}_{{\rm{n}}}}$$

where B_n_ is the background content of the element n in the Gansu soil sample and C_n_ is the measured content of element n in the bus stop dusts. When I_geo_ ≤ 0, the bus stop dusts are non-polluted; when 0 < I_geo_ ≤ 1, the bus stop dusts are slightly to moderately polluted; when 1 < I_geo_ ≤ 2, the bus stop dusts are moderately polluted; when 2 < I_geo_ ≤ 3, the bus stop dusts are moderately to heavily polluted; when 3 < I_geo_ ≤ 4, the bus stop dusts are heavily polluted; when 4 < I_geo_ ≤ 5, the bus stop dusts are heavily to extremely polluted; and when 5 < I_geo_ ≤ 10, the bus stop dusts are extremely polluted^41,42^.

The NSPI can be obtained by the following equation:

2 $${\rm{NSPI}}=\sqrt{\frac{{{\rm{PI}}}_{{\rm{mean}}}^{2}+{{\rm{PI}}}_{{\rm{\max }}}^{2}}{{\rm{2}}}}$$

where PI_max_ and PI_mean_ are the max value and average value of the pollution index (PI) of five harmful elements in every sample. The PI is the ratio of the content of a certain element in the bus stop dusts to the corresponding background value of the element in Gansu soil^43^. When NSPI < 0.7, the bus stop dusts are non-polluted; when 0.7 < NSPI < 1, the bus stop dusts are nearly polluted; when 1 < NSPI < 2, the bus stop dusts are slightly polluted; when 2 < NSPI < 3, the bus stop dusts are moderately polluted; and when NSPI > 3, the bus stop dusts are heavily polluted^40^.
